# Supplementary material for: Evaluation of Cytotoxicity and Metabolic Profiling of Synechocystis sp. Extract Encapsulated in Nano-Liposomes and Nano-Niosomes Using LC-MS, Complemented by Molecular Docking Studies
Source: Biology (Basel). 2024 Jul 31;13(8):581. doi: 10.3390/biology13080581 (PMC11351938; doi:10.3390/biology13080581)
Supplement: Supplementary file 1 [file biology-13-00581-s001.zip › biology-3086604-supplementary.pdf]

Supplementary materials

Table S1: A summary of binding interactions of Erlotinib to the active site of the enzyme with metabolites identified in *Synechocystis* sp. extract

	Distance	Category	From	From Chemistry	To	To Chemistry
1	3.05	Hydrogen Bond;Electrostatic	A:LYS721:NZ	H-Donor	A:GLU738:OE2	H-Acceptor
	3.80	Hydrogen Bond;Electrostatic	A:LYS721:NZ	H-Donor	A:ASP831:OD1	H-Acceptor
	3.16	Hydrogen Bond	A:LYS721:NZ	H-Donor	N:UNK1:O	H-Acceptor
	2.97	Hydrogen Bond	A:LYS721:NZ	H-Donor	N:UNK1:O	H-Acceptor
	2.80	Hydrogen Bond	A:LYS721:NZ	H-Donor	N:UNK1:O	H-Acceptor
	2.02	Hydrogen Bond	N:UNK1:H	H-Donor	A:ASP813:OD2	H-Acceptor
	2.19	Hydrogen Bond	N:UNK1:H	H-Donor	A:ASP813:OD2	H-Acceptor
	2.55	Hydrogen Bond	N:UNK1:H	H-Donor	A:GLU738:OE1	H-Acceptor
	1.95	Hydrogen Bond	N:UNK1:H	H-Donor	N:UNK1:O	H-Acceptor
	2.45	Hydrogen Bond	N:UNK1:H	H-Donor	A:ASP813:OD2	H-Acceptor
	2.22	Hydrogen Bond	N:UNK1:H	H-Donor	A:ASP831:OD1	H-Acceptor
	1.76	Hydrogen Bond	N:UNK1:H	H-Donor	A:ASP831:OD1:B	H-Acceptor
	1.80	Hydrogen Bond	N:UNK1:H	H-Donor	N:UNK1:O	H-Acceptor
	2.92	Hydrogen Bond	N:UNK1:H	H-Donor	N:UNK1:O	H-Acceptor
	2.14	Hydrogen Bond	N:UNK1:H	H-Donor	A:ASP831:OD1	H-Acceptor
	2.41	Hydrogen Bond	N:UNK1:H	H-Donor	A:ASP831:OD1	H-Acceptor
	1.93	Hydrogen Bond	N:UNK1:H	H-Donor	A:ASP831:OD1:B	H-Acceptor
	1.98	Hydrogen Bond	N:UNK1:H	H-Donor	A:LYS851:O	H-Acceptor
	3.70	Hydrogen Bond	N:UNK1:C	H-Donor	N:UNK1:O	H-Acceptor
	3.79	Hydrogen Bond	N:UNK1:C	H-Donor	N:UNK1:O	H-Acceptor
	3.42	Hydrogen Bond	N:UNK1:C	H-Donor	A:ASP831:OD2:B	H-Acceptor
	3.52	Hydrogen Bond	N:UNK1:C	H-Donor	A:ASP813:OD2	H-Acceptor
	2.14	Hydrogen Bond	N:UNK1:H	H-Donor	A:ASP831:OD1	H-Acceptor
	2.41	Hydrogen Bond	N:UNK1:H	H-Donor	A:ASP831:OD1	H-Acceptor
	1.93	Hydrogen Bond	N:UNK1:H	H-Donor	A:ASP831:OD1:B	H-Acceptor
	1.98	Hydrogen Bond	N:UNK1:H	H-Donor	A:LYS851:O	H-Acceptor
	3.52	Hydrogen Bond	N:UNK1:C	H-Donor	A:ASP813:OD2	H-Acceptor
2	3.14	Hydrogen Bond	A:AQ4999:N1	H-Donor	N:UNK1:O	H-Acceptor
	1.95	Hydrogen Bond	N:UNK1:H	H-Donor	N:UNK1:O	H-Acceptor
	2.22	Hydrogen Bond	N:UNK1:H	H-Donor	A:ASP831:OD1	H-Acceptor
	1.76	Hydrogen Bond	N:UNK1:H	H-Donor	A:ASP831:OD1:B	H-Acceptor
	1.80	Hydrogen Bond	N:UNK1:H	H-Donor	N:UNK1:O	H-Acceptor
	2.92	Hydrogen Bond	N:UNK1:H	H-Donor	N:UNK1:O	H-Acceptor
	2.56	Hydrogen Bond	N:UNK1:H	H-Donor	A:ASP831:OD2	H-Acceptor
	2.73	Hydrogen Bond	N:UNK1:H	H-Donor	A:ASP831:OD2	H-Acceptor
	1.96	Hydrogen Bond	N:UNK1:H	H-Donor	A:ARG817:O	H-Acceptor
	3.70	Hydrogen Bond	N:UNK1:C	H-Donor	N:UNK1:O	H-Acceptor

	3.79	Hydrogen Bond	N:UNK1:C	H-Donor	N:UNK1:O	H-Acceptor
	3.42	Hydrogen Bond	N:UNK1:C	H-Donor	A:ASP831:OD2:B	H-Acceptor
	3.35	Hydrogen Bond	N:UNK1:C	H-Donor	N:UNK1:O	H-Acceptor
	3.14	Hydrogen Bond	A:AQ4999:N1	H-Donor	N:UNK1:O	H-Acceptor
	2.56	Hydrogen Bond	N:UNK1:H	H-Donor	A:ASP831:OD2	H-Acceptor
	2.73	Hydrogen Bond	N:UNK1:H	H-Donor	A:ASP831:OD2	H-Acceptor
	1.96	Hydrogen Bond	N:UNK1:H	H-Donor	A:ARG817:O	H-Acceptor
3	2.94	Hydrogen Bond;Electrostatic	A:ARG817:NH1	H-Donor	A:ASP813:OD1	H-Acceptor
	3.06	Hydrogen Bond;Electrostatic	A:ARG817:NH1	H-Donor	A:ASP813:OD2	H-Acceptor
	3.21	Hydrogen Bond;Electrostatic	A:ARG817:NH2	H-Donor	A:ASP813:OD1	H-Acceptor
	2.38	Hydrogen Bond	A:ASN818:ND2	H-Donor	A:ASP813:O	H-Acceptor
	2.02	Hydrogen Bond	N:UNK1:H	H-Donor	A:ASP813:OD2	H-Acceptor
	2.19	Hydrogen Bond	N:UNK1:H	H-Donor	A:ASP813:OD2	H-Acceptor
	1.95	Hydrogen Bond	N:UNK1:H	H-Donor	N:UNK1:O	H-Acceptor
	2.45	Hydrogen Bond	N:UNK1:H	H-Donor	A:ASP813:OD2	H-Acceptor
	2.22	Hydrogen Bond	N:UNK1:H	H-Donor	A:ASP831:OD1	H-Acceptor
	1.76	Hydrogen Bond	N:UNK1:H	H-Donor	A:ASP831:OD1:B	H-Acceptor
	1.80	Hydrogen Bond	N:UNK1:H	H-Donor	N:UNK1:O	H-Acceptor
	2.92	Hydrogen Bond	N:UNK1:H	H-Donor	N:UNK1:O	H-Acceptor
	2.19	Hydrogen Bond	N:UNK1:H	H-Donor	A:ARG817:O	H-Acceptor
	2.71	Hydrogen Bond	N:UNK1:H	H-Donor	A:ASN818:OD1	H-Acceptor
	2.36	Hydrogen Bond	N:UNK1:H	H-Donor	A:ASP831:OD2	H-Acceptor
	2.05	Hydrogen Bond	N:UNK1:H	H-Donor	A:ASN818:OD1	H-Acceptor
	2.68	Hydrogen Bond	N:UNK1:H	H-Donor	A:ASP831:OD1	H-Acceptor
	2.88	Hydrogen Bond	N:UNK1:H	H-Donor	A:ASP831:OD1:B	H-Acceptor
	2.26	Hydrogen Bond	N:UNK1:H	H-Donor	N:UNK1:O	H-Acceptor
	1.58	Hydrogen Bond	N:UNK1:H	H-Donor	N:UNK1:O	H-Acceptor
	2.42	Hydrogen Bond	N:UNK1:H	H-Donor	N:UNK1:O	H-Acceptor
	3.51	Hydrogen Bond	A:AQ4999:C11	H-Donor	N:UNK1:O	H-Acceptor
	3.70	Hydrogen Bond	N:UNK1:C	H-Donor	N:UNK1:O	H-Acceptor
	3.79	Hydrogen Bond	N:UNK1:C	H-Donor	N:UNK1:O	H-Acceptor
	3.42	Hydrogen Bond	N:UNK1:C	H-Donor	A:ASP831:OD2:B	H-Acceptor
	3.44	Hydrogen Bond	N:UNK1:C	H-Donor	A:ARG817:O	H-Acceptor
	3.97	Electrostatic	N:UNK1:P	Positive	A:ASP813:OD2	Negative
	4.05	Electrostatic	N:UNK1:P	Positive	A:ASP831:OD1	Negative
	3.59	Electrostatic	N:UNK1:P	Positive	A:ASP831:OD1:B	Negative
	2.19	Hydrogen Bond	N:UNK1:H	H-Donor	A:ARG817:O	H-Acceptor
	2.71	Hydrogen Bond	N:UNK1:H	H-Donor	A:ASN818:OD1	H-Acceptor
	2.36	Hydrogen Bond	N:UNK1:H	H-Donor	A:ASP831:OD2	H-Acceptor
	2.05	Hydrogen Bond	N:UNK1:H	H-Donor	A:ASN818:OD1	H-Acceptor
	2.68	Hydrogen Bond	N:UNK1:H	H-Donor	A:ASP831:OD1	H-Acceptor
	2.88	Hydrogen Bond	N:UNK1:H	H-Donor	A:ASP831:OD1:B	H-Acceptor
	2.42	Hydrogen Bond	N:UNK1:H	H-Donor	N:UNK1:O	H-Acceptor

4	2.02	Hydrogen Bond	N:UNK1:H	H-Donor	A:ASP813:OD2	H-Acceptor
	2.19	Hydrogen Bond	N:UNK1:H	H-Donor	A:ASP813:OD2	H-Acceptor
	1.95	Hydrogen Bond	N:UNK1:H	H-Donor	N:UNK1:O	H-Acceptor
	2.45	Hydrogen Bond	N:UNK1:H	H-Donor	A:ASP813:OD2	H-Acceptor
	2.22	Hydrogen Bond	N:UNK1:H	H-Donor	A:ASP831:OD1	H-Acceptor
	1.76	Hydrogen Bond	N:UNK1:H	H-Donor	A:ASP831:OD1:B	H-Acceptor
	1.80	Hydrogen Bond	N:UNK1:H	H-Donor	N:UNK1:O	H-Acceptor
	2.92	Hydrogen Bond	N:UNK1:H	H-Donor	N:UNK1:O	H-Acceptor
	2.59	Hydrogen Bond	N:UNK1:H	H-Donor	A:ASP813:OD2	H-Acceptor
	2.88	Hydrogen Bond	N:UNK1:H	H-Donor	N:UNK1:O	H-Acceptor
	2.23	Hydrogen Bond	N:UNK1:H	H-Donor	A:ASP813:OD2	H-Acceptor
	2.00	Hydrogen Bond	N:UNK1:H	H-Donor	A:ASP831:OD1	H-Acceptor
	1.84	Hydrogen Bond	N:UNK1:H	H-Donor	A:ASP831:OD1:B	H-Acceptor
	3.40	Hydrogen Bond	A:GLY833:CA	H-Donor	N:UNK1:O	H-Acceptor
	3.54	Hydrogen Bond	A:GLY833:CA	H-Donor	N:UNK1:O	H-Acceptor
	3.69	Hydrogen Bond	A:GLY833:CA	H-Donor	N:UNK1:O	H-Acceptor
	3.70	Hydrogen Bond	N:UNK1:C	H-Donor	N:UNK1:O	H-Acceptor
	3.79	Hydrogen Bond	N:UNK1:C	H-Donor	N:UNK1:O	H-Acceptor
	3.42	Hydrogen Bond	N:UNK1:C	H-Donor	A:ASP831:OD2:B	H-Acceptor
	2.59	Hydrogen Bond	N:UNK1:H	H-Donor	A:ASP813:OD2	H-Acceptor
	2.88	Hydrogen Bond	N:UNK1:H	H-Donor	N:UNK1:O	H-Acceptor
	2.23	Hydrogen Bond	N:UNK1:H	H-Donor	A:ASP813:OD2	H-Acceptor
	2.00	Hydrogen Bond	N:UNK1:H	H-Donor	A:ASP831:OD1	H-Acceptor
	1.84	Hydrogen Bond	N:UNK1:H	H-Donor	A:ASP831:OD1:B	H-Acceptor
	3.69	Hydrogen Bond	A:GLY833:CA	H-Donor	N:UNK1:O	H-Acceptor
5	3.80	Hydrogen Bond;Electrostatic	A:LYS721:NZ	H-Donor	A:ASP831:OD1	H-Acceptor
	2.94	Hydrogen Bond;Electrostatic	A:ARG817:NH1	H-Donor	A:ASP813:OD1	H-Acceptor
	3.06	Hydrogen Bond;Electrostatic	A:ARG817:NH1	H-Donor	A:ASP813:OD2	H-Acceptor
	3.21	Hydrogen Bond;Electrostatic	A:ARG817:NH2	H-Donor	A:ASP813:OD1	H-Acceptor
	3.16	Hydrogen Bond	A:LYS721:NZ	H-Donor	N:UNK1:O	H-Acceptor
	2.97	Hydrogen Bond	A:LYS721:NZ	H-Donor	N:UNK1:O	H-Acceptor
	2.80	Hydrogen Bond	A:LYS721:NZ	H-Donor	N:UNK1:O	H-Acceptor
	2.02	Hydrogen Bond	N:UNK1:H	H-Donor	A:ASP813:OD2	H-Acceptor
	2.19	Hydrogen Bond	N:UNK1:H	H-Donor	A:ASP813:OD2	H-Acceptor
	1.95	Hydrogen Bond	N:UNK1:H	H-Donor	N:UNK1:O	H-Acceptor
	2.45	Hydrogen Bond	N:UNK1:H	H-Donor	A:ASP813:OD2	H-Acceptor
	2.22	Hydrogen Bond	N:UNK1:H	H-Donor	A:ASP831:OD1	H-Acceptor
	1.76	Hydrogen Bond	N:UNK1:H	H-Donor	A:ASP831:OD1:B	H-Acceptor
	1.80	Hydrogen Bond	N:UNK1:H	H-Donor	N:UNK1:O	H-Acceptor
	2.92	Hydrogen Bond	N:UNK1:H	H-Donor	N:UNK1:O	H-Acceptor
	3.40	Hydrogen Bond	A:GLY833:CA	H-Donor	N:UNK1:O	H-Acceptor
	3.54	Hydrogen Bond	A:GLY833:CA	H-Donor	N:UNK1:O	H-Acceptor

	3.70	Hydrogen Bond	N:UNK1:C	H-Donor	N:UNK1:O	H-Acceptor
	3.79	Hydrogen Bond	N:UNK1:C	H-Donor	N:UNK1:O	H-Acceptor
	3.42	Hydrogen Bond	N:UNK1:C	H-Donor	A:ASP831:OD2:B	H-Acceptor
	4.12	Hydrogen Bond	A:ARG817:NH2	H-Donor	A:TRP856	Pi-Orbitals
	2.80	Hydrogen Bond	A:LYS721:NZ	H-Donor	N:UNK1:O	H-Acceptor
	2.45	Hydrogen Bond	N:UNK1:H	H-Donor	A:ASP813:OD2	H-Acceptor
	2.22	Hydrogen Bond	N:UNK1:H	H-Donor	A:ASP831:OD1	H-Acceptor
	1.76	Hydrogen Bond	N:UNK1:H	H-Donor	A:ASP831:OD1:B	H-Acceptor
	2.92	Hydrogen Bond	N:UNK1:H	H-Donor	N:UNK1:O	H-Acceptor
	3.54	Hydrogen Bond	A:GLY833:CA	H-Donor	N:UNK1:O	H-Acceptor
	3.42	Hydrogen Bond	N:UNK1:C	H-Donor	A:ASP831:OD2:B	H-Acceptor
	4.43	Hydrophobic	A:CYS773	Alkyl	N:UNK1	Alkyl
	3.96	Hydrophobic	A:ARG817	Alkyl	N:UNK1	Alkyl
	4.40	Hydrophobic	A:ARG817	Alkyl	N:UNK1	Alkyl
	4.68	Hydrophobic	A:ARG817	Alkyl	N:UNK1	Alkyl
	4.08	Hydrophobic	A:LYS889	Alkyl	N:UNK1	Alkyl
	5.26	Hydrophobic	A:PRO890	Alkyl	N:UNK1	Alkyl
	4.78	Hydrophobic	N:UNK1	Alkyl	A:LEU775	Alkyl
	5.11	Hydrophobic	A:TRP856	Pi-Orbitals	N:UNK1	Alkyl
7	3.09	Hydrogen Bond	N:UNK1:H	H-Donor	N:UNK1:O	H-Acceptor
	2.65	Hydrogen Bond	N:UNK1:H	H-Donor	N:UNK1:O	H-Acceptor
	2.09	Hydrogen Bond	N:UNK1:H	H-Donor	N:UNK1:Cl	H-Acceptor
	3.37	Hydrogen Bond	A:LYS721:NZ	H-Donor	N:UNK1:O	H-Acceptor
	2.45	Hydrogen Bond	N:UNK1:H	H-Donor	A:ASP813:OD2	H-Acceptor
	4.41	Hydrophobic	A:VAL702	Alkyl	N:UNK1	Alkyl
	5.22	Hydrophobic	A:PHE699	Pi-Orbitals	N:UNK1	Alkyl
	3.77	Hydrophobic	A:PHE699	Pi-Orbitals	N:UNK1	Alkyl
	4.79	Hydrophobic	A:PHE699	Pi-Orbitals	N:UNK1	Alkyl
	4.55	Hydrophobic	A:AQ4999	Pi-Orbitals	N:UNK1	Alkyl
	5.27	Hydrophobic	A:AQ4999	Pi-Orbitals	N:UNK1:C	Alkyl
	5.35	Hydrophobic	A:AQ4999	Pi-Orbitals	N:UNK1:C	Alkyl
8	3.80	Hydrogen Bond;Electrostatic	A:LYS721:NZ	H-Donor	A:ASP831:OD1	H-Acceptor
	2.94	Hydrogen Bond;Electrostatic	A:ARG817:NH1	H-Donor	A:ASP813:OD1	H-Acceptor
	3.06	Hydrogen Bond;Electrostatic	A:ARG817:NH1	H-Donor	A:ASP813:OD2	H-Acceptor
	3.21	Hydrogen Bond;Electrostatic	A:ARG817:NH2	H-Donor	A:ASP813:OD1	H-Acceptor
	3.00	Hydrogen Bond	A:LYS721:NZ	H-Donor	N:UNK1:O	H-Acceptor
	3.27	Hydrogen Bond	A:ARG817:NH1	H-Donor	N:UNK1:O	H-Acceptor
	2.65	Hydrogen Bond	N:UNK1:H	H-Donor	N:UNK1:O	H-Acceptor
	2.09	Hydrogen Bond	N:UNK1:H	H-Donor	N:UNK1:Cl	H-Acceptor
	2.86	Hydrogen Bond	N:UNK1:H	H-Donor	A:ASP831:OD1:B	H-Acceptor
	3.02	Hydrogen Bond	N:UNK1:H	H-Donor	A:ASP831:OD2:B	H-Acceptor
	3.30	Hydrogen Bond	N:UNK1:C	H-Donor	A:ASP813:OD2	H-Acceptor

	3.64	Hydrogen Bond	N:UNK1:C	H-Donor	A:ASP831:OD1	H-Acceptor
	3.16	Hydrogen Bond	N:UNK1:C	H-Donor	A:ASP831:OD1:B	H-Acceptor
9	2.80	Hydrogen Bond	A:LYS855:N	H-Donor	A:HOH4:O	H-Acceptor
	2.09	Hydrogen Bond	N:UNK1:H	H-Donor	N:UNK1:Cl	H-Acceptor
	4.80	Hydrophobic	A:PRO853	Alkyl	N:UNK1	Alkyl
	5.29	Hydrophobic	A:PRO853	Alkyl	N:UNK1	Alkyl
	4.58	Hydrophobic	A:LYS855	Alkyl	N:UNK1	Alkyl
	4.78	Hydrophobic	N:UNK1:C	Alkyl	A:PRO853	Alkyl
	4.92	Hydrophobic	A:PHE699	Pi-Orbitals	N:UNK1	Alkyl
	4.95	Hydrophobic	A:TRP856	Pi-Orbitals	N:UNK1	Alkyl
	5.46	Hydrophobic	A:TRP856	Pi-Orbitals	N:UNK1	Alkyl
10	2.09	Hydrogen Bond	N:UNK1:H	H-Donor	N:UNK1:Cl	H-Acceptor
	4.12	Hydrogen Bond	A:ARG817:NH2	H-Donor	A:TRP856	Pi-Orbitals
	3.90	Hydrophobic	N:UNK1:C	C-H	A:PHE699	Pi-Orbitals
	5.38	Hydrophobic	A:LEU775	Alkyl	N:UNK1	Alkyl
	4.97	Hydrophobic	A:ARG817	Alkyl	N:UNK1	Alkyl
	4.88	Hydrophobic	A:PRO853	Alkyl	N:UNK1	Alkyl
	5.45	Hydrophobic	A:LYS855	Alkyl	N:UNK1	Alkyl
	5.34	Hydrophobic	A:LYS889	Alkyl	N:UNK1	Alkyl
	4.47	Hydrophobic	A:LYS889	Alkyl	N:UNK1	Alkyl
	4.81	Hydrophobic	A:PRO890	Alkyl	N:UNK1	Alkyl
	4.78	Hydrophobic	A:TRP856	Pi-Orbitals	N:UNK1	Alkyl
	5.08	Hydrophobic	A:AQ4999	Pi-Orbitals	N:UNK1	Alkyl
11	2.09	Hydrogen Bond	N:UNK1:H	H-Donor	N:UNK1:Cl	H-Acceptor
	4.84	Hydrophobic	A:ARG817	Alkyl	N:UNK1	Alkyl
	4.30	Hydrophobic	A:PHE699	Pi-Orbitals	N:UNK1	Alkyl
12	3.09	Hydrogen Bond	A:LYS721:NZ	H-Donor	N:UNK1:O	H-Acceptor
	2.09	Hydrogen Bond	N:UNK1:H	H-Donor	N:UNK1:Cl	H-Acceptor
	3.09	Hydrogen Bond	A:LYS721:NZ	H-Donor	N:UNK1:O	H-Acceptor
	4.62	Hydrophobic	A:VAL702	Alkyl	N:UNK1	Alkyl
	4.85	Hydrophobic	A:CYS773	Alkyl	N:UNK1	Alkyl
	5.41	Hydrophobic	A:CYS773	Alkyl	N:UNK1	Alkyl
	4.10	Hydrophobic	A:ARG817	Alkyl	N:UNK1	Alkyl
	4.76	Hydrophobic	A:PHE699	Pi-Orbitals	N:UNK1	Alkyl
	3.73	Hydrophobic	A:PHE699	Pi-Orbitals	N:UNK1	Alkyl
	4.63	Hydrophobic	A:AQ4999	Pi-Orbitals	N:UNK1	Alkyl
13	2.09	Hydrogen Bond	N:UNK1:H	H-Donor	N:UNK1:Cl	H-Acceptor
	2.86	Hydrogen Bond	N:UNK1:H	H-Donor	A:ASP831:OD1:B	H-Acceptor
	3.02	Hydrogen Bond	N:UNK1:H	H-Donor	A:ASP831:OD2:B	H-Acceptor
	2.09	Hydrogen Bond	N:UNK1:H	H-Donor	A:ASP831:OD1	H-Acceptor

	2.62	Hydrogen Bond	A:ARG817:CD	H-Donor	N:UNK1:O	H-Acceptor
	2.09	Hydrogen Bond	N:UNK1:H	H-Donor	A:ASP831:OD1	H-Acceptor
	2.62	Hydrogen Bond	A:ARG817:CD	H-Donor	N:UNK1:O	H-Acceptor
	4.58	Hydrophobic	A:LEU834	Alkyl	N:UNK1	Alkyl
	4.45	Hydrophobic	A:PRO853	Alkyl	N:UNK1	Alkyl
	4.72	Hydrophobic	N:UNK1:C	Alkyl	A:PRO853	Alkyl
	3.83	Hydrophobic	A:PHE699	Pi-Orbitals	N:UNK1	Alkyl
	5.30	Hydrophobic	A:AQ4999	Pi-Orbitals	N:UNK1	Alkyl
14	2.80	Hydrogen Bond	A:LYS855:N	H-Donor	A:HOH4:O	H-Acceptor
	2.30	Hydrogen Bond	N:UNK1:H	H-Donor	A:ASP776:OD1	H-Acceptor
	2.09	Hydrogen Bond	N:UNK1:H	H-Donor	N:UNK1:Cl	H-Acceptor
	2.86	Hydrogen Bond	N:UNK1:H	H-Donor	A:ASP831:OD1:B	H-Acceptor
	3.02	Hydrogen Bond	N:UNK1:H	H-Donor	A:ASP831:OD2:B	H-Acceptor
	1.82	Hydrogen Bond	N:UNK1:H	H-Donor	A:LYS851:O	H-Acceptor
	4.12	Hydrogen Bond	A:ARG817:NH2	H-Donor	A:TRP856	Pi-Orbitals
	2.30	Hydrogen Bond	N:UNK1:H	H-Donor	A:ASP776:OD1	H-Acceptor
	2.09	Hydrogen Bond;Halogen	N:UNK1:H	H-Donor;Halogen Acceptor	N:UNK1:Cl	H-Acceptor;Halogen
	2.86	Hydrogen Bond	N:UNK1:H	H-Donor	A:ASP831:OD1:B	H-Acceptor
	3.02	Hydrogen Bond	N:UNK1:H	H-Donor	A:ASP831:OD2:B	H-Acceptor
	1.82	Hydrogen Bond	N:UNK1:H	H-Donor	A:LYS851:O	H-Acceptor
	4.97	Electrostatic	A:ARG817:NH1	Positive	N:UNK1	Pi-Orbitals
	5.10	Hydrophobic	A:VAL702	Alkyl	N:UNK1	Alkyl
	4.53	Hydrophobic	A:PRO853	Alkyl	N:UNK1	Alkyl
	5.27	Hydrophobic	A:PRO853	Alkyl	N:UNK1	Alkyl
	4.45	Hydrophobic	A:LYS855	Alkyl	N:UNK1	Alkyl
	3.97	Hydrophobic	A:LYS889	Alkyl	N:UNK1	Alkyl
	5.13	Hydrophobic	A:PRO890	Alkyl	N:UNK1	Alkyl
	4.72	Hydrophobic	N:UNK1:Cl	Alkyl	A:LEU820	Alkyl
	5.43	Hydrophobic	A:PHE699	Pi-Orbitals	N:UNK1	Alkyl
	5.26	Hydrophobic	A:PHE699	Pi-Orbitals	N:UNK1	Alkyl
	5.35	Hydrophobic	A:TRP856	Pi-Orbitals	N:UNK1	Alkyl
	5.20	Hydrophobic	A:TRP856	Pi-Orbitals	N:UNK1	Alkyl
	4.61	Hydrophobic	A:AQ4999	Pi-Orbitals	N:UNK1	Alkyl
	5.09	Hydrophobic	A:AQ4999	Pi-Orbitals	N:UNK1:Cl	Alkyl
15	3.01	Hydrogen Bond	A:ALA858:N	H-Donor	A:HOH1:O	H-Acceptor
	2.52	Hydrogen Bond	A:SER871:OG	H-Donor	A:HOH1:O	H-Acceptor
	2.61	Hydrogen Bond	A:SER875:OG	H-Donor	A:TRP856:O	H-Acceptor
	2.57	Other	N:UNK1:Mg	Metal	A:TRP856:O	H-Acceptor
	2.64	Other	N:UNK1:Mg	Metal	A:SER871:O	H-Acceptor
	2.31	Other	N:UNK1:Mg	Metal	A:SER875:OG	H-Acceptor
	3.08	Other	N:UNK1:Mg	Metal	A:HOH1:O	H-Acceptor
16	3.17	Hydrogen Bond	A:ILE735:N	H-Donor	A:ALA731:O	H-Acceptor

	3.22	Hydrogen Bond	A:ILE735:N	H-Donor	N:UNK1:O	H-Acceptor
	2.88	Hydrogen Bond	N:UNK1:H	H-Donor	A:ALA731:O	H-Acceptor
	3.22	Hydrogen Bond	A:ILE735:N	H-Donor	N:UNK1:O	H-Acceptor
	2.88	Hydrogen Bond	N:UNK1:H	H-Donor	A:ALA731:O	H-Acceptor
	4.86	Hydrophobic	A:CYS773	Alkyl	N:UNK1	Alkyl
	3.75	Hydrophobic	A:ARG817	Alkyl	N:UNK1	Alkyl
	4.94	Hydrophobic	N:UNK1:C	Alkyl	A:LEU834	Alkyl
	5.33	Hydrophobic	A:PHE699	Pi-Orbitals	N:UNK1	Alkyl
	4.17	Hydrophobic	A:PHE699	Pi-Orbitals	N:UNK1	Alkyl
17	3.01	Hydrogen Bond	A:ALA858:N	H-Donor	A:HOH1:O	H-Acceptor
	2.52	Hydrogen Bond	A:SER871:OG	H-Donor	A:HOH1:O	H-Acceptor
	2.61	Hydrogen Bond	A:SER875:OG	H-Donor	A:TRP856:O	H-Acceptor
	2.57	Other	N:UNK1:Mg	Metal	A:TRP856:O	H-Acceptor
	2.64	Other	N:UNK1:Mg	Metal	A:SER871:O	H-Acceptor
	2.31	Other	N:UNK1:Mg	Metal	A:SER875:OG	H-Acceptor
	3.08	Other	N:UNK1:Mg	Metal	A:HOH1:O	H-Acceptor
18	4.50	Hydrophobic	A:VAL702	Alkyl	N:UNK1	Alkyl
	4.77	Hydrophobic	A:CYS773	Alkyl	N:UNK1	Alkyl
	3.93	Hydrophobic	A:ARG817	Alkyl	N:UNK1	Alkyl
	3.70	Hydrophobic	A:PHE699	Pi-Orbitals	N:UNK1	Alkyl
	4.86	Hydrophobic	A:PHE699	Pi-Orbitals	N:UNK1	Alkyl
	4.71	Hydrophobic	A:AQ4999	Pi-Orbitals	N:UNK1	Alkyl
19	3.80	Hydrogen Bond;Electrostatic	A:LYS721:NZ	H-Donor	A:ASP831:OD1	H-Acceptor
	2.94	Hydrogen Bond;Electrostatic	A:ARG817:NH1	H-Donor	A:ASP813:OD1	H-Acceptor
	3.06	Hydrogen Bond;Electrostatic	A:ARG817:NH1	H-Donor	A:ASP813:OD2	H-Acceptor
	3.21	Hydrogen Bond;Electrostatic	A:ARG817:NH2	H-Donor	A:ASP813:OD1	H-Acceptor
	3.27	Hydrogen Bond	A:LYS721:NZ	H-Donor	N:UNK1:O	H-Acceptor
	1.89	Hydrogen Bond	N:UNK1:H	H-Donor	A:ASP813:OD2	H-Acceptor
	3.27	Hydrogen Bond	A:LYS721:NZ	H-Donor	N:UNK1:O	H-Acceptor
	1.89	Hydrogen Bond	N:UNK1:H	H-Donor	A:ASP813:OD2	H-Acceptor
	4.60	Hydrophobic	A:VAL702	Alkyl	N:UNK1	Alkyl
	4.36	Hydrophobic	A:CYS773	Alkyl	N:UNK1	Alkyl
	5.15	Hydrophobic	A:CYS773	Alkyl	N:UNK1	Alkyl
	4.80	Hydrophobic	A:ARG817	Alkyl	N:UNK1	Alkyl
	3.75	Hydrophobic	A:PHE699	Pi-Orbitals	N:UNK1	Alkyl
	5.06	Hydrophobic	A:PHE699	Pi-Orbitals	N:UNK1	Alkyl
	4.65	Hydrophobic	A:AQ4999	Pi-Orbitals	N:UNK1	Alkyl
20	3.80	Hydrogen Bond;Electrostatic	A:LYS721:NZ	H-Donor	A:ASP831:OD1	H-Acceptor
	2.94	Hydrogen Bond;Electrostatic	A:ARG817:NH1	H-Donor	A:ASP813:OD1	H-Acceptor

	3.06	Hydrogen Bond;Electrostatic	A:ARG817:NH1	H-Donor	A:ASP813:OD2	H-Acceptor
	3.21	Hydrogen Bond;Electrostatic	A:ARG817:NH2	H-Donor	A:ASP813:OD1	H-Acceptor
	3.27	Hydrogen Bond	A:LYS721:NZ	H-Donor	N:UNK1:O	H-Acceptor
	1.89	Hydrogen Bond	N:UNK1:H	H-Donor	A:ASP813:OD2	H-Acceptor
	3.27	Hydrogen Bond	A:LYS721:NZ	H-Donor	N:UNK1:O	H-Acceptor
	1.89	Hydrogen Bond	N:UNK1:H	H-Donor	A:ASP813:OD2	H-Acceptor
	4.60	Hydrophobic	A:VAL702	Alkyl	N:UNK1	Alkyl
	4.36	Hydrophobic	A:CYS773	Alkyl	N:UNK1	Alkyl
	5.15	Hydrophobic	A:CYS773	Alkyl	N:UNK1	Alkyl
	4.80	Hydrophobic	A:ARG817	Alkyl	N:UNK1	Alkyl
	3.75	Hydrophobic	A:PHE699	Pi-Orbitals	N:UNK1	Alkyl
	5.06	Hydrophobic	A:PHE699	Pi-Orbitals	N:UNK1	Alkyl
	4.65	Hydrophobic	A:AQ4999	Pi-Orbitals	N:UNK1	Alkyl
21	3.80	Hydrogen Bond;Electrostatic	A:LYS721:NZ	H-Donor	A:ASP831:OD1	H-Acceptor
	2.94	Hydrogen Bond;Electrostatic	A:ARG817:NH1	H-Donor	A:ASP813:OD1	H-Acceptor
	3.06	Hydrogen Bond;Electrostatic	A:ARG817:NH1	H-Donor	A:ASP813:OD2	H-Acceptor
	3.21	Hydrogen Bond;Electrostatic	A:ARG817:NH2	H-Donor	A:ASP813:OD1	H-Acceptor
	3.27	Hydrogen Bond	A:LYS721:NZ	H-Donor	N:UNK1:O	H-Acceptor
	3.16	Hydrogen Bond	A:LYS721:NZ	H-Donor	N:UNK1:O	H-Acceptor
	2.97	Hydrogen Bond	A:LYS721:NZ	H-Donor	N:UNK1:O	H-Acceptor
	1.89	Hydrogen Bond	N:UNK1:H	H-Donor	A:ASP813:OD2	H-Acceptor
	2.02	Hydrogen Bond	N:UNK1:H	H-Donor	A:ASP813:OD2	H-Acceptor
	2.19	Hydrogen Bond	N:UNK1:H	H-Donor	A:ASP813:OD2	H-Acceptor
	3.27	Hydrogen Bond	A:LYS721:NZ	H-Donor	N:UNK1:O	H-Acceptor
	1.89	Hydrogen Bond	N:UNK1:H	H-Donor	A:ASP813:OD2	H-Acceptor
	4.60	Hydrophobic	A:VAL702	Alkyl	N:UNK1	Alkyl
	4.36	Hydrophobic	A:CYS773	Alkyl	N:UNK1	Alkyl
	5.15	Hydrophobic	A:CYS773	Alkyl	N:UNK1	Alkyl
	4.80	Hydrophobic	A:ARG817	Alkyl	N:UNK1	Alkyl
	3.75	Hydrophobic	A:PHE699	Pi-Orbitals	N:UNK1	Alkyl
	5.06	Hydrophobic	A:PHE699	Pi-Orbitals	N:UNK1	Alkyl
	4.65	Hydrophobic	A:AQ4999	Pi-Orbitals	N:UNK1	Alkyl
22	3.59	Electrostatic	A:ASP831:OD2	Negative	N:UNK1	Pi-Orbitals
	4.57	Electrostatic	A:ASP831:OD1:B	Negative	N:UNK1	Pi-Orbitals
	3.70	Hydrophobic	N:UNK1:C	C-H	A:PHE699	Pi-Orbitals
	5.30	Hydrophobic	A:PHE699	Pi-Orbitals	N:UNK1	Pi-Orbitals
	5.64	Hydrophobic	A:AQ4999	Pi-Orbitals	N:UNK1	Pi-Orbitals
	4.97	Hydrophobic	A:LEU834	Alkyl	N:UNK1	Alkyl
	4.27	Hydrophobic	N:UNK1:C	Alkyl	A:PRO853	Alkyl
